# Logic, inference, understanding: cross-domain generalization for generative language models

**DOI:** 10.3389/frai.2026.1800372

**Published:** 2026-06-16

**Authors:** Rasmus Blanck, Bill Noble

**Affiliations:** 1 Department of Philosophy, Linguistics and Theory of Science, Centre for Linguistic Theory and Studies in Probability, University of Gothenburg, Gothenburg, Sweden; 2 MIT Media Lab, Center for Constructive Communication, Massachusetts Institute of Technology, Cambridge, MA, United States

**Keywords:** formal semantics, generalization, language models, logic, natural language inference

## Abstract

Neural systems for Natural Language Inference (NLI) have seen impressive performance over the last ten years, but their ability to generalize beyond their training data has repeatedly been questioned. The NLI task has long been considered as a proxy for the wider problem of Natural Language Understanding (NLU), implicitly motivated by relying on an inferentialist conception of semantics. This paper draws on insights from work in formal logic and semantics to introduce distinctions between different notions of generalizations (in-domain vs. cross-domain, and linguistic vs. inferential) in an attempt to disentangle the problem of generalization. We leverage the theoretical contributions in experiments addressing the inferential generalization power of autoregressive NLI models.

## 1 Introduction

How do we know if a computational model or system *understands* natural language? Finding a satisfying way to answer this question is an important challenge in computational linguistics and central to the Natural Language Understanding (NLU) subject area. For certain applications, text classification can provide a sufficient answer. The NLU component of traditional dialogue systems, for example, classifies user utterances as expressing one of several pre-defined *intents*: Does the user intend to *inquire about a train schedule, book an itinerary*, or *modify an existing itinerary*? While such an approach can work well in task-oriented dialogue systems, the circumscribed conversational domain and limited nuance in model outputs makes it insufficient for evaluating human-like language understanding more generally.

Looking to theoretical linguistics, *possible world semantics* is probably the most influential theory of natural language semantics. It relates the meaning of a sentence to the set of *possible worlds* — precisely those worlds in which the sentence is true. The meaning of a sentence like *the Moon is made of cheese* thus equates to the set of possible worlds in which the Moon is indeed made of cheese. Except in a rather artificially constrained possibility space,^1^ it is hard to imagine a machine learning task that assesses NLU by relating sentences to something as intangible (and infinitary) as sets of possible worlds. Inference tasks like RTE and NLI are a way around this problem.^2^ These tasks challenge a model to classify the inferential relationship between a pair of sentences (often: a *premise* and *hypothesis*) among a small set of possible labels (e.g., *entailment, contradiction* or *neutral*).

It has long been acknowledged that NLI is a necessary condition for NLU (Condoravdi et al., 2003; MacCartney, 2009). That NLI is also *sufficient* for NLU is sometimes explicitly stated, for example by Goldberg as “solving [the natural language inference task] perfectly entails human level understanding of language” (Goldberg, 2017, p. 141). Another illuminating quote to this end is: “The recognition of textual entailment is without doubt one of the ultimate challenges for any NLP system: if it is able to do so with reasonable accuracy, it is clearly an indication that it has some thorough understanding of how language works” (Bos and Markert, 2005, p. 628).

More often, the sufficiency of NLI for NLU is just implicitly assumed, as shown through the following samples from central NLI papers published during the NLI heyday:

- “[…] NLI is an ideal testing ground for theories of semantic representation […]” (Bowman et al., 2015, p. 640);
- “[…] natural language understanding models […] are evaluated on the task of natural language inference” (Nangia et al., 2017, p. 1);
- “The SNLI dataset […] [is] explicitly constructed in order to require understanding sentence semantics.” (Conneau et al., 2017, p. 671);
- “[NLI] […] has emerged as a practical test bed for work on sentence understanding.” (Conneau et al., 2018, p. 2475);
- “NLI has been proposed as a benchmark task for natural language understanding […]” (Naik et al., 2018, p. 2340);
- “[NLI] is well positioned to serve as a benchmark task for research on NLU.” (Williams et al., 2018, p. 1112);
- The paper by Nie et al. (2020) is titled *Adversarial NLI: A New Benchmark for Natural Language Understanding*.

Secondary evidence is provided by (Madaan et al., 2025) who write “In the recent past, a popular way of evaluating natural language understanding (NLU), was to consider a model's ability to perform natural language inference (NLI) tasks” (Madaan et al., 2025, p. 9229). All in all, it seems fair to say that the most influential NLI papers are operating under the assumption that NLU can be evaluated by performance on NLI tasks.

The underlying intuition that classification of inferential relationships has any bearing on NLU can be understood as adopting a version of *inferentialist semantics*, wherein the meaning of a linguistic expression is determined by its *inferential role* — that which can be inferred from its use in a given situation. Indeed, Bowman, in his PhD thesis, explicitly acknowledges basing his account of sentence meaning on inferentialism: “I focus on an alternative framing of sentence meaning that selects inferential relationships […] as the primary element of meaning. […] This framing is based in the inferentialist […] approach to formal semantics […]” (Bowman, 2016, p. 7). In contrast with theories of meaning that center language–world relations, inferentialist theories center language–language relations (Steinberger and Murzi, 2017). From an NLP perspective, this allows for a tractable NLU task that is nevertheless comprehensive and theoretically grounded.

Central to successful NLU, especially when NLI is taken as a proxy for it, is the ability for models to generalize beyond their training data. It is well established that NLI systems generally see drastic performance drops when evaluated on other datasets, or other out-of-distribution data. The aim of this paper is to address this problem of generalization both theoretically and experimentally. The theoretic contributions concern firstly the interpretation of the NLI and RTE inference labels, and their relationship to abstract logical consequence relations. Secondly, we introduce two distinctions — *cross-domain* vs. *in-domain* and *linguistic* vs. *inferential* generalizations — to start disentangling the problem of generalization for NLI and NLU, and highlight how training data may limit the generalization ability of a trained classifier. The experimental part reports on results from experiments testing the generalization power of autoregressive generative language models. The experiments are set up in a way as to focus on the impact of inferential, rather than linguistic, generalization.

**This paper** is organized as follows. Section 2 reviews the theoretical foundations for the rest of the paper, and Section 3 discusses the NLI and RTE relations. Section 4 dissects the problem of generalization. Section 5 discusses potential issues with fine-tuning for NLI. Section 6 reports on experiments testing the generalization abilities of generative language models.

## 2 Theoretical background

### 2.1 Inferentialism and its relation to NLI and NLU

Steinberger and Murzi (2017) and Incurvati and Schlöder (2024) both distinguish two central theses of inferentialism. The first, that we here call (M) (for meaning), states that the semantic value of a linguistic expression is determined by its inferential role. The other, (U) (for understanding), states that to understand a linguistic expression is to know its inferential role. Incurvati and Schlöder (2024, p. 38) further identify the semantic thesis (S) that the semantic value of an expression *is* its inferential role.

By contrast, a traditional truth-conditional conception of meaning entails that the meaning of an expression is its truth conditions (in parallel to (S)), and that to know the meaning is to know what those truth conditions are (in parallel to (U)) (Davidson, 1967).

We show that the claim that NLI is sufficient for NLU implies the assumption (U). Suppose that NLI is sufficient for NLU, and, for a contradiction, that knowing the inferential role of an expression is not sufficient for understanding it. There is then an expression such that understanding it goes beyond knowing its inferential role. But then even perfect performance on NLI, which involves exactly knowing the inferential roles of expressions, does not suffice for understanding that particular expression. Hence NLI is not sufficient for NLU, contradicting the assumption. It follows that claiming that NLI is sufficient for NLU implies implicitly adopting the inferentialist assumption (U).

The assumption (U) would in turn seem to rest on (M): Suppose that understanding an expression is knowing its inferential role, but that its inferential role does not determine its meaning. Then it is possible to have understanding of an expression without its meaning being determinate, or there being some other component of meaning that is not necessary to grasp in order to understand the expression. Both seem far fetched.

There are two more distinctions on inferentialism that have bearing on NLI and the problem of generalization, the first being local vs. global; essentially the question of whether inferentialism should be applicable to only restricted portions of language (such as logical, moral, or theoretical expressions), or to language as a whole (Steinberger and Murzi, 2017, p. 201). The version of inferentialism that implicitly underpins NLI is a global one: we are not only interested in inferences in, e.g., the moral or logical domain, but in natural language as a whole.

The other is which stance to take on semantic atomism/molecularism/holism. Semantic atomism argues that the meaning of an expression is independent of the meaning of other expressions, and is incompatible with inferentialism (Steinberger and Murzi, 2017, p. 201). Semantic holism is the position that the meaning of an expression depends on those of every other expression, and seems untenable in relation to NLI/NLU. To understand an expression on a holistic perspective would involve having knowledge of every inference in which it is involved (Steinberger and Murzi, 2017, p. 201).

Semantic molecularism provides some middle ground, where only the relation to meanings of certain other expressions are relevant for determining the meaning of the expression. Brandom (2007) explains molecularism as taking sentences as the principled object of analysis. By analyzing the (inferential) relationships on a given corpus of sentences, molecular inferentialism may ascribe semantic values also to constituents of those sentences, that can be used to generalize to a wider class of sentences (Brandom, 2007, p. 671). This perspective seems entirely compatible with the larger project of NLI.

In this light, the theoretical connection between NLI and inferentialist semantics that seems to be underlying, e.g., the discussion of Bowman et al. (2015, Section 3.3.) on how NLI performance can be used to evaluate the quality of sentence embeddings as carriers of meaning can be summarized as follows:

1. The meaning of a sentence *S* in a language L is determined by its inferential role, which is, or is at least strongly related to, the set of sentences entailed by *S*:
$$\begin{array}{l} \left[\left[S\right]\right]=\left\{S^{\prime }\in L|S⊨S^{\prime }\right\}. \end{array}$$
2. *Understanding the meaning* of sentence in L is evidenced by performance in computing membership in [[*S*]] for an arbitrary *S*. If we characterize *language understanding* as understanding arbitrary sentences of a given language, this is (by currying) equivalent to performance in determining whether or not *P* ⊨ *H* for a pair of sentences *P* and *H*.
3. A neural network sentence encoder M is an algorithm that computes a function from sentences to real-valued vector representations of meaning:
$$M:L\to {\mathbb{R}}^{n}\text{.}$$
4. The extent to which M produces good representations of meaning for L can be approximated by the performance of a classifier trained to determine the entailment relation between pairs of sentence representations
$$C:{\mathbb{R}}^{n}\times {\mathbb{R}}^{n}\text{.}$$

Importantly, one does not need to be a full-fledged philosophical inferentialist to think that classifying the entailment relation between pairs of sentences is an effective indicator of understanding: It is possible to reject (S), and for example subscribe to truth-conditional possible-world semantics, while still accepting (U) and (M) (Incurvati and Schlöder, 2024, p. 42).

In possible world semantics, the meaning of a sentence is identified with the set of possible worlds in which it is true. The ability to classify arbitrary pairs of sentences is then *functionally equivalent* to identifying the set of possible worlds in which it is true, modulo the ability of the language to discern between possible worlds.

### 2.2 Object–metalanguage distinction

In developing the semantic conception of truth, Tarski (1933) famously introduced the distinction between object language and metalanguage. In short, the object language can be understood as the language being described or studied, and the metalanguage as the language used to describe or study the object language. What makes this point crucial is Tarski's theorem on the undefinability of truth, which proves that no semantically closed formal language can define an adequate notion of truth for itself. By extension, any notion of logical consequence for a language that is based on the notion of truth for the same language must be defined in a richer metalanguage. In practice, the metalanguage is often chosen as an extension of the object language, for example by enriching the object language with symbols or notions used to discuss and interpret the expressions of the object language.

While, in general, inference need not be primarily based on the notion of truth, both truth and falsity feature prominently in descriptions of the NLI labels. Consider, for example, the instructions provided to annotators in the process of creating the influential SNLI dataset:

We will show you the caption for a photo. We will not show you the photo. Using only the caption and what you know about the world:

- Write one alternate caption that is **definitely** a **true** description of the photo. […]
- Write one alternate caption that **might be** a **true** description of the photo. […]
- Write one alternate caption that is **definitely** a **false** description of the photo. […]

(Bowman et al., 2015, p. 634, emphasis in original)

The SNLI inference labels are therefore explicitly based on a notion of truth. This means that if we believe (as some authors do) that the *E* label of NLI can be expressed as the propositional formula *a* → *b*, then it must be made clear whether this is an expression in the object language, which is often some fragment of English, or in the metalanguage. Similar considerations apply to other notational devices such as *a* ⊨ *b*, *Eab*, etc.

If we treat *a* → *b* as an expression of the object language, we must assume either that → is a symbol that can be used to form new expressions from other expressions, so that the object language is, at least in part, symbolic, or that it is used as shorthand for some natural language expression such as “if … then …”. Its semantics can then be defined in the metalanguage, for example by “*b* must be true whenever *a* is true”, or “there is a construction that converts a proof of *a* to a proof of *b*”, or some other suitable notion of consequence.

By contrast, we may treat *a* → *b* as an expression in the metalanguage, stating that a certain relationship holds between *a* and *b*. This is the approach most commonly taken in relation to NLI, where the enriched metalanguage vocabulary contains symbols such as *E*, *C* and *N*, so that we can write expressions like *Eab* to denote that *a* entails *b*, and define the semantics of *E* in the metalanguage.^3^

### 2.3 Abstract consequence relations

A consequence relation is a relation between sets of premises Γ and conclusions ϕ. Given a set of sentences *X* we may think of a consequence relation ⊨ on *X* as a subset of the set P(X)×X, where P(X) is the powerset of *X*. This is inspired by the abstract algebraic view of logic (see, e.g. Font, 2016). On this abstract view, there is a multitude of different consequence relations for a given collection of sentences, and we may ask what different properties these various consequence relations may have. In light of the discussion in the previous section, we treat *X* as the object language, and ⊨ as a metalanguage relation between object-language sentences.

By contrast, for “traditional”, formal, consequence relations, such as consequence in classical propositional logic, we are assuming some closure properties on the language *X*, such that it is closed under the usual propositional connectives.^4^ For example, if *p* is in the language *X*, then ¬*p* better be too. In the classical propositional case, we may further assume that the relation between *p* and ¬*p* is a special one, governed by the bivalent truth-table for the connective ¬ that can be specified in the metalanguage. Typically, there is also a formally defined semantics underlying the consequence relation, that is defined by structural induction over the logical symbols of the language. In the applications considered below, such closure properties, semantic properties and inductive definitions may not in general be assumed. Rather, we may regard the degree to which a learned consequence relation satisfies such properties as a proxy for the generalization problem (see, e.g., Gubelmann et al., 2024).

A consequence relation is finitary if, whenever Γ ⊨ ϕ, there is a finite Δ ⊆ Γ s.t. Δ ⊨ ϕ. This is akin to the compactness theorem for first-order logic. We restrict attention to finitary consequence relations, since an infinitary consequence relation may need to use infinitely many premises to reach a conclusion, which seems implausible for consequence relations over natural language expressions. This means that finitary consequence relations over an object language that can express conjunction can be regarded as subsets of *X* × *X*, that is, as binary relations between sentences. We simply replace a finite set of premises with the conjunction of the premises, since {*p*_1_, …, *p*_*n*_} ⊨ ϕ iff (*p*_1_ ∧ (… ∧ *p*_*n*_)…) ⊨ ϕ.

There is a large collection of concepts that can be used to motivate a claim that a sentence pair (*a, b*) is an entailment. These involve as different relations as lexical entailment, monotonicity of determiners, inductive inference, shallow heuristics and formal consequence of various kinds. Among these is the special case where *a* entails *b* because there are formalizations α and β in some logic *L* of *a* and *b* such that α entails β according to the consequence relation of that logic: α ⊨_*L*_ β. Importantly, different logics may make different verdicts on whether α entails β or not. Hence, there can be no master notion of deductive entailment that faithfully emulates all of these notions. An abstract consequence relation that is not generated by some formal logic but rather constructed from data must bundle all of these relations together.^5^ Such a consequence relation, however, must take a stance even for the pairs where the verdicts of different logics may differ, which makes it possible to gauge which notion of deductive validity the relation is compatible with by checking its classification of suitably chosen sentence pairs.

## 3 The NLI and RTE relations

Natural Language Inference (NLI) and Recognizing Textual Entailment (RTE) have been centerpieces of natural language processing over the last 20 years. In short, the two tasks have in common to determine whether or not a sentence (the hypothesis) can be inferred from another sentence (NLI: premise; RTE: text). RTE classification comes in two flavours: Either a two-way classification (entailment/non-entailment) or a three-way classification (entailment/contradiction/unknown) whereas NLI is a three-way classification problem (entailment/contradiction/neutral). The three-way RTE classification is closely aligned with — but not exactly identical to — the NLI classification, and it is easy to see that two-way RTE is a special case of three-way RTE: Simply group contradiction and unknown together as non-entailment.

It is not clear in the literature exactly what notion of entailment is being considered in the respective tasks, or whether or not those notions can be expressed as logical consequence, or deductive validity, in some formal logic. The NLI entailment relation is best understood as deductive as seen in the instructions given to the crowd workers (“…definitely a true description …” (Bowman et al., 2015, p. 634; see also Gubelmann et al., 2024), whereas the RTE relation is less strict: “cases in which inference is very probable (but not completely certain) are still judged as True” (RTE-1; Dagan et al., 2006, p. 183) and “a human reading T would infer that H is most likely true” (RTE-5; (TAC2009, 2009)).

Against this background, we will now relate NLI and RTE classification to each other, and also to the abstract consequence relations discussed in Section 2.3. The output of the classifier can be seen as an approximation of a consequence relation in the following way: If the classifier classifies the pair of a premise *a* and a hypothesis *b* as an entailment, then the corresponding pair (*a, b*) is a member of the learned approximation of the consequence relation. We write this as *a* ⊨ *b*. The corresponding NLI/RTE classification is written *Eab* in the cases where it needs to be differentiated from the consequence relation. Similarly, we may use a given abstract consequence relation ⊨ to define, in the metalanguage, a target notion of NLI/RTE entailment by


Eab iff a⊨b.

Having established how to understand the entailment relation as an abstract consequence relation, we turn to its relationship to the other NLI relations. Following, among others, MacCartney and Manning (2014), we define


Cab iff a⊨¬b.

Note that this definition requires that the object language can express negation: There must be some way to turn a sentence *b* into its negation ¬*b*. In English, this can be achieved uniformly by prepending the sentence with “It is not the case that …”. The semantics of this expression can then be defined in the metalanguage. Finally, we let


Nab iff a⊭b & a⊭¬b,

with the intended reading that the pair (*a, b*) is neither an entailment nor a contradiction. This is entirely in line with, for example, the instructions for constructing the MNLI dataset, stating that neutral means that “neither condition [entailment or contradiction] applies” (Williams et al., 2018). This definition furthermore requires negation and conjunction in the metalanguage. Hence, if both the object language and the metalanguage can express negation and conjunction, then all the three NLI labels can be reconstructed entirely from the binary consequence relation (or equivalently, expressed in the metalanguage entirely in terms of the *E* label together with object- and metalanguage negation, and metalanguage conjunction).

For a similar application, we show that two- and three-way RTE classification is essentially the same task for object languages being able to express negation. Two-way RTE classifies sentence pairs into entailment and non-entailment, whereas three-way RTE further divides non-entailment into contradiction and unknown. First, it is obvious that two-way RTE is just a special case of three-way RTE where contradiction and unknown are grouped together into non-entailment.

Secondly, we show how to reconstruct three-way RTE from two-way RTE: If a pair (*p, h*) is labeled entailment in the two-way classification, then label it entailment in the three-way classification too. If the pair is labeled non-entailment, then check the classification of (*p*, ¬*h*). This requires negation in the object language. If this pair is labeled entailment in the two-way classification, then label the original pair (*p, h*) contradiction in the three-way classification. Finally, if (*p*, ¬*h*) is too labeled non-entailment, then label the original pair unknown in the three-way classification. Note that this means that the entailment relation completely determines the other RTE relations.

On a related note, Gubelmann et al. (2024) argue that “careful qualitative inspection shows clearly that it [the RTE challenge dataset of Wang et al. (2018)] represents a deductive notion of inference” (p. 26). This reveals a discrepancy between the RTE entailment relation as described in the RTE guidelines and the entailment relation as encoded in the RTE challenge dataset — a point we return to in Section 4.2. If RTE does indeed encode a deductive notion of inference, and if it can be further established to be equivalent to the NLI entailment relation, it then follows that the RTE and NLI tasks are equivalent in their entirety, since all the NLI relations can be uniquely determined from the RTE entailment relation, and vice versa.

## 4 The problem of generalization

The problem of generalization for models trained on NLI datasets is widely recognized (see, e.g., Talman and Chatzikyriakidis (2019); McCoy et al. (2020); Gubelmann et al. (2024); Madaan et al. (2025) for some data points a few years apart). The purpose of this section is to add some conceptual clarity to the problem of generalization. To this end, we introduce two distinctions as grounds for the further discussion.

The first distinction is *in-domain* versus *cross-domain* generalization. In-domain generalization concerns generalizing to unseen combinations of sentences from the training distribution, approximating some external consequence relation [as discussed in Section 2.3], or learning meta-inferential properties of the learned consequence relation (as in Blanck et al. (2025)). Cross-domain, or out-of-dataset, generalization is, for example, training on one NLI dataset and testing on another, or generalizing to previously unseen sentences.

The other distinction is *linguistic* versus *inferential* generalization. Linguistic generalization pertains to generalizing to unseen linguistic material. Inferential generalization instead concerns the ability of the classifier to pick up on properties of the inference relation itself, such as meta-inferential principles, or features that are otherwise hard to explicitly express in the training data due to certain notions not being available in the object language.

These distinctions are independent, but not uncorrelated.^6^ For example, successful cross-domain generalization in a setting where a classifier is trained (or fine-tuned) on one dataset and evaluated on another typically involves being successful both in linguistic and inferential generalization. Completely separating these aspects would seem to require a strictly controlled and carefully devised experimental setup.

### 4.1 Linguistic generalization

NLI datasets are based on some fragment of a natural language such as English.^7^ The vocabulary of the dataset is limited and, typically, not all combinations of sentences from the relevant fragment feature as premise–hypothesis pairs in the dataset. The dataset is further split into training and test sets. This means that an NLI classifier is typically trained only on a smaller part of a subset of a fragment of the language. Thus, for purposes of discussing linguistic generalization, we need to consider a range of collections of sentence pairs, from smaller to larger:

1. Sentence pairs occurring in the training set;
2. sentence pairs occurring in the full data set (training + validation + test);
3. sentence pairs constructed from sentences occurring in the full data set;
4. the collection of pairs of declarative sentences that can be constructed from the fragment of English sufficient to construct all sentences in the dataset;
5. pairs of arbitrary declarative English sentences.

To take NLI classification as a proxy for the much broader task of NLU then rests on a four-tiered linguistic generalization problem. First, to generalize from the collection of sentence pairs in the training set (1) to the pairs in the test set (2). Assuming that the data is split into training and test only after the full dataset has been constructed, annotation artifacts are going to be present in both sets. The success of this generalization is measured through the usual evaluation metrics. Secondly, to generalize to new combinations of sentences from the dataset (3). This keeps the total collection of sentences fixed, but creates new pairs for example by swapping premises and hypotheses as in Wang et al. (2019b). If the model has fully captured the meaning of the sentences *p* and *h*, then classifying the pair (*h, p*) should be no more difficult than classifying (*p, h*). The coherence of this mode of generalization can be gauged through meta-inferential properties and internal consistency (see, e.g., Minervini and Riedel (2018); Li et al. (2019); Wu and Last (2025); Srikanth and Rudinger (2025); Blanck et al. (2025)). These are both in-domain generalizations.

Thirdly, to generalize further to the whole relevant fragment of English (4). This can be seen as fixing a vocabulary and formation rules and generating a new collection of sentences based on that vocabulary. Essentially, this would amount to keeping the lexical part of the semantics fixed but extending the semantics compositionally. Finally, to extend the classification to the entirety of English (5). For a model to have achieved full NLU competence, it should be able to classify the inference relation for *any* sentence pair, not just pairs drawn from the original dataset or the limited language fragment sufficient to generate it. These two latter modes correspond to cross-domain generalization such as testing on a dataset not used for training, and are commonly measured through the usual evaluation metrics.

Yet another issue is the distribution of labels over data. Datasets like SNLI (Bowman et al., 2015), MNLI (Williams et al., 2018) and HANS (McCoy et al., 2019) are balanced. But this does not seem to be the case for the collection of arbitrary pairs of declarative sentences. Two declarative sentences chosen at random are likely to have little to do with each other, and should most likely then be labeled as neutral. A further possible complication is that the truth value of many sentences can not be evaluated out of context (consider, e.g., examples like “He is here”).

In this light, there is a lot of ground for a plain NLI classifier to cover if we expect it to be able to generalize from the limited collection of sentence pairs in the training set to arbitrary sentence pairs in the language under discussion, if only for the sheer difference in vocabulary size. Larger, pretrained models are likely to perform better when it comes to linguistic generalization, due to their generally better linguistic capabilities. The question remains whether these better linguistic generalization abilities also extends to inferential generalization.

### 4.2 Inferential generalization

While a key motivation of NLI is to evaluate the NLU capability of models on arbitrary English sentences, it has been observed that the most performant NLI models do not generalize well outside the distribution of their training sets. This has been blamed on annotation artifacts in the datasets and the models using shallow heuristics (see, e.g., Gururangan et al., 2018; Poliak et al., 2018; McCoy et al., 2019; Gubelmann et al., 2022). Beyond revealing annotation artifacts, Gubelmann et al. (2024) acknowledge that the failure of cross-domain generalization could be due to the different datasets encoding different notions of inference.^8^ They distinguish between inductive and deductive modes of reasoning, where an example of an inductively correct argument is

(P) The streets are wet. (H) It has rained.
      (Gubelmann et al., 2024, p. 22)

Models trained or fine-tuned on datasets of this kind may struggle on test sets containing deductive modes of reasoning, where their main example is formal reasoning such as Aristotelian syllogisms (Gubelmann et al., 2024). This raises the question of whether poor cross-domain generalization can be attributed to incomprehensive NLU (as in the case of relying on shallow heuristics) or differences in the logical paradigm implicit in different inference datasets.

A similar observation is made in some detail by Kalouli et al. (2023), which they have also experimentally confirmed. They re-annotate parts of the SICK dataset (Marelli et al., 2014) using two different annotation guidelines with one taking a strictly logical stance, and the other using common-sense reasoning. In this way, the sentence pair “A woman is squeezing a lemon”/“The woman is squeezing juice out of a lemon” may be assigned different labels under the two annotation schemes: *N* according to pure logic (it is possible to squeeze a lemon without getting any juice out of it), but *E* under the common-sense approach (but we usually don't do that to lemons). In this way, they arrive at three different versions of SICK literally encoding different entailment relations, since some of the labels (roughly 9%) in the reannotation differ from the original.

Cross-domain performance reported by Kalouli et al. (2023) for BERT and RoBERTa models trained on the three versions are SICK are generally good across the full test sets, with cross-domain performance drops in the rough range of 2–7 percentage points, and in-domain performance comparable to that of BERT and RoBERTa on SNLI and MNLI. However, when evaluated specifically on pairs where re-annotation labels differ from the original ones, performance drops are in the range of 40–70 percentage points. Moreover, when evaluated on pairs where the logic stance and the common-sense stance yield different labels, in-domain performance is generally much lower (no model surpassing 50%) with cross-domain performance drops of around 10 percentage points. They draw the conclusion that this kind of pairs are especially hard (both for human annotators and trained systems), and that models model a mix of logical and common-sense reasoning, and therefore fail to distinguish between these when explicitly tested for just one notion of inference. Since the linguistic material is kept fixed across their three versions of SICK, the only remaining explanation would seem to be the different notions of entailment encoded through the different labels of the re-annotations.

### 4.3 Abstract consequence relations revisited

Given a fixed collection of sentences *X*, recall that we can identify a logic over this language with its consequence relation, and further identify this with a subset of *X* × *X*. As a consequence, we may compare different logics over the same language by comparing whether certain sentence pairs are in the respective logics or not. For example, when we take this perspective on the consequence relation of classical propositional logic over a language being able to express conjunction and negation, it is going to contain all sentence pairs (*a* ∧ ¬*a, b*) for all sentences *a, b* in the language. The reason is that *a* ∧ ¬*a* is a contradiction in classical logic for any choice of *a*, and that this logic is explosive — meaning that each sentence follows from a contradiction. The same is not going to hold for paraconsistent logics, since they are not explosive. Similarly, classical logic is going to contain the pairs (*a, b* ∨ ¬*b*) for all sentences *a, b*, since the law of excluded middle is a classical validity. By contrast, intuitionistic logic is not going to contain all such pairs, as the law of the excluded middle is not intuitionistically valid.

This perspective can now be leveraged as a new mode of evaluation for inferential generalization, and also for evaluation of the suitability of training data (see Blanck et al., 2025 for a precursor of this perspective). Though it seems unlikely that an abstract consequence relation learned from data is going to align completely with any previously studied formal consequence relation, such a formal relation can be used as a point of comparison in evaluating the learned relation and also help decide which properties to probe for. Suppose, for example, that our external logical intuitions align with classical propositional logic, and that we want our NLI model to be compatible with this consequence relation. This has as a consequence, among others, that contradictions are symmetric, so that the formal consequence relation is closed under the meta-inferential rule *Cab* → *Cba* (suitably formulated in terms of entailment). An abstract consequence relation approximated by a successfully trained classifier should therefore too be governed by this meta-inference.

Data of this explicit form is generally not going to be in the training data, since both *C* and → are typically expressions in the metalanguage rather than in the object language. However, if the model is able to pick up on the salient properties of the inference relation from the training data, as opposed to just learning shallow heuristics, it should also be able to generalize from classifying a pair (*a, b*) as a contradiction to also classifying the pair (*b, a*) as a contradiction. If the model's approximation is closed under this generalization, it seems reasonable to say that it has fully captured this particular in-domain inferential generalization, and that this property of the inference relation is encoded in the dataset. If we want our model to be compatible with classical propositional logic as a whole, there is of course a wide range of other inferential generalizations to be tested. Should we find that the model consistently fails to learn the desired meta-inferential principles from the training, it could imply that the data is not rich enough to encode the desired mode of inference.

This kind of evaluation also lends itself to cross-domain generalizations. Suppose that we have two classifiers α and β, having seen the same pretraining (if any), differing only in that α is trained/fine-tuned on a dataset *A* while β is trained/fine-tuned on *B*. If α and β satisfy different meta-inferential principles, or do so to different degrees, then we must conclude that α and β are not equivalent. If we further assume that *A* and *B* are based on the same linguistic material, only organized differently in the different datasets, we must conclude that the two datasets encode different notions of entailment.^9^ Hence, bringing the object/metalanguage distinction to the forefront, and coupling it with abstract consequence relations, gives a way to disentangle the problem of generalization in a meaningful and experimentally testable way.

### 4.4 Internal consistency

There is a collection of papers evaluating the *internal* or *self-consistency* of language models and NLI systems, e.g., Kassner et al. (2021); Mitchell et al. (2022); Nakamura et al. (2023); Parcalabescu and Frank (2024); Srikanth and Rudinger (2025). A language model may be regarded as internally inconsistent if it, for example, produces answers that do not go together, such as “Eagles are mammals” and “Eagles lay eggs” (example adapted from Kassner et al., 2021) modulo some background knowledge and a reasoning system. In this example, the background knowledge could be “Mammals do not lay eggs” and the reasoning system could be classical first-order logic, which would produce a contradiction together with the two premises.

There are at least two issues bearing on this. The first is related to concepts such as the closed-world assumption, pragmatic relevance, and coreference.^10^ For example, each SNLI premise is an image caption taken from the Flickr30k corpus (Young et al., 2014). An implicit assumption is that the caption accurately describes the image, or at least that it exhaustively enough describes the salient features of the scene. Typically, a sentence pair like “A dog is running”/“A dog is sleeping” would be labeled as a contradiction, based on this assumption: The premise mentions exactly one dog, and we may therefore rule out that there are other dogs in the image, so that the dog in the hypothesis must be the same as the previous one. But this sentence pair becomes a contradiction in a logical sense only if paired with additional assumptions such as “There is exactly one dog”.

The other issue is that consistency can be defined in a number of ways, for example:

1. *X* is consistent if *X* ⊭ ⊥, where ⊥ is a designated propositional symbol intended as a necessary falsehood;
2. *X* is consistent if there is a formula ϕ such that *X* ⊭ ϕ;
3. *X* is consistent if there is no formula ϕ such that *X* ⊨ ϕ and *X* ⊨ ¬ϕ.

While these three definitions are equivalent for classical logic, they come apart for other logics. Furthermore, a set *X* that is consistent over some logic may fail to be consistent over another. This suggests strongly that to be able to coherently evaluate the self-consistency of a language model, we must make explicit which notion of consistency is being considered, as well as the notion of consequence on which that notion of consistency is based. For NLI systems in particular, being trained or fine-tuned on a particular dataset, it is imperative to be clear on whether the notion of inference being encoded by the examples in the dataset is the same as the external notion of consequence being used to assess the self-consistency.

## 5 Fine-tuning for NLI

As articulated by Bowman et al. (2015) and summarized in Section 2.1 of this paper, inference tasks can be seen as a way to *probe the representation space* of encoder models. In particular, if a model produces representations that can be separated into *entailments* and *non-entailments* by a classifier, we can infer that the representations encode the information required for NLU. Allowing the full model to be fine-tuned on the inference dataset increases the risk that good performance is reflective of the model's ability to learn annotation artifacts, rather than make use of semantically rich representations, as discussed in Section 4.2. This suggests that the proper way to use an inference task to assess the NLU is to use the pre-trained model as a feature extractor and train a simple classifier on top of those representations.

On the other hand, it could be argued that treating such a model as merely a feature extractor fails to assess its true NLU potential, since it is not given the chance to attend to semantic features that are particularly relevant to the notion of inference encoded in the dataset. Indeed, within a year of the original BERT release (Devlin et al., 2019), it was observed that BERT performs markedly better (in terms of in-domain generalization) on NLI and other tasks when encoder layers are allowed to be fine-tuned (Peters et al., 2019).

A parallel difficulty extends to generative models: Since there is generally no canonical method for extracting sentence-level representations from these models,^11^ probing their NLU with a simple classifier is not so straight-forward. A more natural way to test generative language models is to set up the inference task as a multiple choice question and assess the model based on the label to which it assigns the most probability. Here again we are faced with a dilemma: If we test the pre-trained model in a zero-shot fashion, poor performance may be reflective of insufficient information about the task (the answer format, the notion of inference, etc.), rather than poor NLU. On the other hand, just as with encoder models, fine-tuning introduces the possibility of learning unwanted dataset biases.^12^

With this in mind, comparing inference task performance under different conditions can be used to obtain a more robust NLU assessment. One might consider, for example,

1. performance of the pre-trained model with no fine-tuning;
2. performance of the model *only trained on the train set* (i.e., with no pre-training); and
3. performance of the pre-trained and fine-tuned model.

The first of these conditions can be realized in at least two ways, the first of which is more natural for encoder models, while the later makes more sense for generative models:

- probing classifier performance with no base model fine-tuning; and
- zero-shot performance in a multiple-choice setup.

If we see better in-domain generalization (i.e., better test set performance) in condition (3) compared to (2), we can infer that the pre-trained model captures linguistic meaning sufficiently to, for example, make inferences with sentence constructions and lexical items not attested in the training set. Likewise, better performance in condition (3) compared to (1) can indicate that the model needs fine-tuning to understand the task, or it could indicate that (3) is learning unwanted dataset artifacts. Further targeted experiments, such as using a *hypothesis-only baseline* (Gururangan et al., 2018; Poliak et al., 2018), can help to give more insight here.

## 6 NLI generalization in autoregressive language models

Autoregressive language models have received a great deal of attention in the last several years, due in large part to their use in text generation applications, such as chatbots. Anecdotally, these models demonstrate an impressive degree of language understanding; they appear to generalize well, even in situations with very little in-domain training data. It is notable, however, that even in the age of ubiquitous generative LLMs with parameters on the order tens of billions, transformer encoder models augmented with specialized attention mechanisms and training schemes remain competitive on standard NLI datasets like SNLI. For example, the top two scoring models on the official SNLI leaderboard, Sun et al. (2020) (acc 92.3%) and Wang et al. (2021) (acc. 93.1%) meet this description.^13^ This observation is not unique to NLI — it a pattern that can be seen across a variety of token- and sentence-level classification tasks (Qorib et al., 2024; Benayas et al., 2025; Zhang et al., 2025) especially when parameter counts are taken into account (Weller et al., 2026).

While published parameter counts for the more recent models are hard to come by, they are likely a few orders of magnitude higher than those of BERT-like models. Brown et al. (2020) report that the few-shot performance of GPT-3, a 175B-parameter model, is slightly worse on the SuperGLUE RTE benchmark (Wang et al., 2019a) than a BERT-large (340M) baseline at 71.7%. This is far below RoBERTa-large (355M) performance in the mid-to-high 80s (Liu et al., 2019). For a further comparison, Radford et al. (2018) report an accuracy on SNLI of 89.9% for fine-tuned early GPT models, which was state-of-the-art performance at the time, but subsequently surpassed by BERT-like encoder models. The zero-shot performance of GPT-3.5 Turbo on SNLI is reported at 64.66% (Ye et al., 2023), brought up to 82.20% in a few-shot setting (Chen et al., 2023). Liu et al. (2023) report accuracy scores for ChatGPT (3.5 Turbo) and GPT-4 on the MNLI development set, scoring 55.40% and 64.08%, respectively. They compare these results with a RoBERTa baseline scoring at 90.02%.

Additionally, in recent years, researchers have retreated from inference tasks for assessing generalized NLU, preferring large conglomerated benchmarks that test a variety of different capabilities (e.g., Li et al., 2024). Nevertheless, Madaan et al. (2025) argue that NLI is a useful task for assessing generative models, since it discriminates well among different model sizes and stages of training and that, moreover, there is still room for improvement on certain NLI benchmarks. The close relationship that inference tasks have to theoretical linguistic semantics is another reason to continue using it to assess NLU for generative models.

In this study, we use NLI to investigate the effect that model size and pre-training data have on cross-domain generalization for moderately-sized generative language models. Talman and Chatzikyriakidis (2019) found that encoder models (both BiLSTMs and BERT) demonstrate systematic failures in cross-domain NLI generalization on the SNLI, MNLI, and SICK datasets; reporting, for example, a cross-domain performance drop of around 30 percentage points for models trained on SNLI or MNLI when evaluated on SICK. Similar results were obtained by Kalouli et al. (2023). They fine-tuned BERT and RoBERTa models on SICK, as well as on re-annotated versions of SICK where purely logical and common-sense reasoning had been disambiguated. These models were then tested on SNLI and MNLI. The performance drop was of the same order of magnitude (30–40 percentage points) as that reported by Talman and Chatzikyriakidis (2019). Following this methodology, we investigate the both in-domain and cross-domain generalization of similarly-sized generative language models.

It is well-known that SNLI in particular suffers from annotation artifacts that affect model performance, and that are revealed by comparing hypothesis-only models with majority class baselines (Gururangan et al., 2018; Poliak et al., 2018; Tsuchiya, 2018). To clarify the role of those biases in cross-domain generalization, we also perform smaller scale hypothesis-only cross-domain experiments.

### 6.1 Experimental setup

We experiment with Pythia (Biderman et al., 2023), a family of decoder-only transformer-based language models trained on the Pile (Gao et al., 2020), a publicly available collection of English-language datasets. Pythia models are available in a variety of sizes but maintain the exact same training scheme (batch size, sequence length, and data shuffling) for every model, which allows us to disentangle the effects of parameter count and quantity of pre-training data. The basic architecture of Pythia is similar to GPT-3 (Brown et al., 2020) but incorporates recent well-established modeling improvements such as rotary embeddings (see Biderman et al. (2023, §2.3) for details^14^). Larger sizes of Pythia are achieved by scaling the number of layers, embedding dimension size, and number of attention heads at ratios that maintain parity with popular commercial and closed-source models (see Table 1).

**Table 1:** Pythia model sizes used in this paper.

Model size	Non-embedding params	Layers	Model dim	Heads
14M	1,189,888	6	128	4
70M	18,915,328	6	512	8
160M	85,056,000	12	768	12
410M	302,311,424	24	1024	16

See (Biderman et al., 2023, Table 1) for further details.

**Figure 1:**
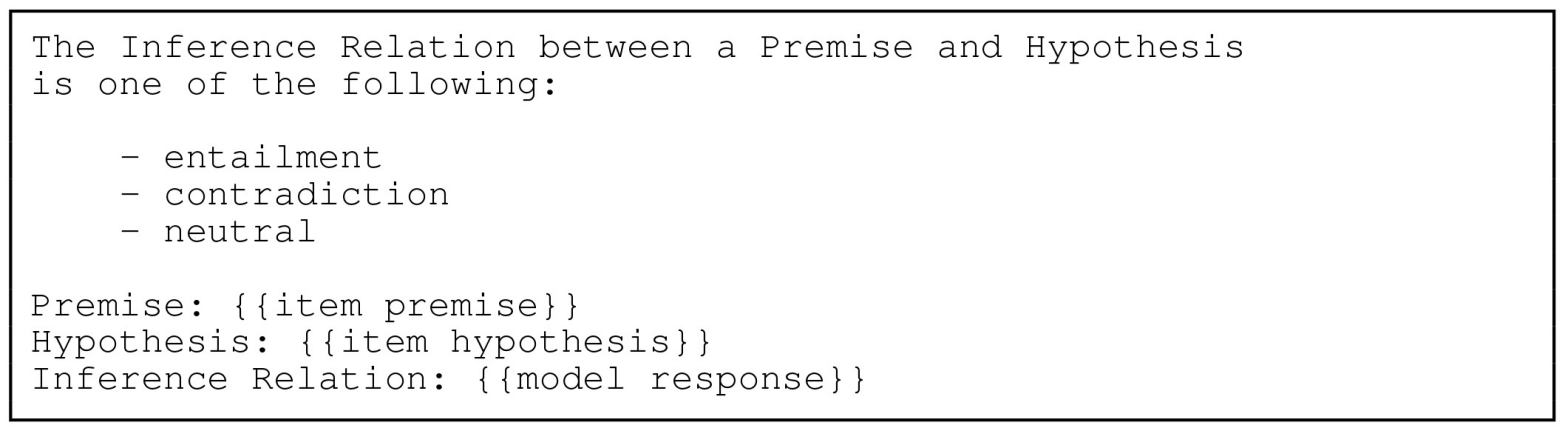
NLI prompt used for training and testing Pythia models.

In our experiment, we test models with 14, 70, 160, 410 million parameters^15^ and four different checkpoints in the pre-training process: 10K, 50K, 100K, and 143K training steps, the last of which represents all of the available pre-training data. To adapt the task to the generative setting, we prompt the model to generate one of the three NLI labels, given the premise–hypothesis pair, as shown in Figure 1.

All models were fine-tuned with average token-level cross-entropy loss between the generated tokens (including stop token) and expected output, according to the string representation of the ground-truth training item labels.^16^ Models were fine-tuned for a maximum of 10 epochs, with checkpoints every 500 steps. The checkpoint with the smallest in-domain development set loss was used for testing.

### 6.2 Datasets

We experimented with two different NLI datasets. SNLI (Bowman et al., 2015) is a large crowd-sourced NLI dataset with approximately 550K training pairs. It was collected by taking image captions as premises eliciting hypothesis sentences for each of the three inference labels from a crowd worker. The captions are taken from the Flickr30k dataset (Young et al., 2014) which was constructed by crowd workers following the annotation guidelines of Hodosh et al. (2013). SICK (Marelli et al., 2014) is a much smaller dataset at just 4439 training items. It uses normalized image captions and video descriptions as premises, and the hypotheses are constructed by a collection of lexical and syntactic manipulations. The SICK image captions originate from the Flickr8k dataset Hodosh et al., (2013), again constructed by crowd workers following the annotation guidelines of (Hodosh et al. 2013). The video description premises are taken from the SemEval 2012 STS MSR-Video Description data set (Agirre, 2012), originally collected as described by Chen and Dolan (2011). Premises were paired up with their own manipulated sentences, as well as with sentences originating from unrelated premises. The premise–hypothesis pairs are then annotated for entailment relations by crowd workers.

While Talman and Chatzikyriakidis (2019) experimented not only on SNLI and SICK, but also on MNLI, their results show less cross-domain performance degradation between SNLI and MNLI than between SICK and either of MNLI/SNLI. Moreover, due to the relatively small size of SICK, they did not use it for training of their models. Kalouli et al. (2023) did fine-tune on SICK and test on both SNLI and MNLI, but not the other way around. In this light, a comparison between SNLI and SICK on generative transformer models, with SICK also used in fine-tuning, seem like the more relevant minimal comparison in order to see to which extent the results of Talman and Chatzikyriakidis (2019) and Kalouli et al. (2023) generalize to this class of models.

### 6.3 Results and analysis

**Figure 2:**
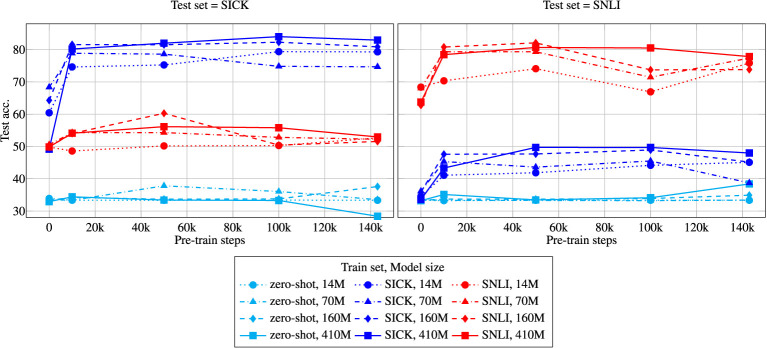
NLI accuracy (%) by number of pre-training steps for different model sizes, showing zero-shot, in-domain, and cross-domain performance for the SNLI and SICK datasets. See Biderman et al. (2023) for pre-training details.

The results are summarized in Figure 2, which shows the effect of pre-training on the y-axis, and Table 2, which shows in-domain and cross-domain performance for the best models, analogous to the analysis of Talman and Chatzikyriakidis (2019, Table 4).

**Table 2:** Test accuracies (% recall) for each of the three NLI labels and macro-average thereof.

Train	Test	E	C	N	Macro avg.	Δ	Size	Steps
zero-shot zero-shot zero-shot zero-shot	SNLI SNLI SNLI SNLI	0.0 97.3 69.2 44.0	0.0 0.9 11.4 70.7	100.0 2.4 24.1 0.5	33.3 33.5 34.9 38.4		14M 70M 160M 410M	143000 0 143000 143000
zero-shot zero-shot zero-shot zero-shot	SICK SICK SICK SICK	99.1 0.1 73.1 97.7	2.5 15.9 17.3 1.0	0.0 97.4 22.3 4.5	33.9 37.8 37.6 34.4		14M 70M 160M 410M	0 50000 143000 10000
SNLI SNLI SNLI SNLI	SNLI SNLI SNLI SNLI	84.1 85.5 87.1 86.4	74.4 80.3 81.5 78.7	69.0 72.2 77.7 76.9	75.8 79.3 82.1 80.7		14M 70M 160M 410M	143000 10000 50000 50000
SNLI SNLI SNLI SNLI	SICK SICK SICK SICK	82.0 87.7 86.0 86.7	47.6 48.9 61.7 49.0	28.4 26.3 33.1 32.7	52.6 54.3 60.3 56.1	-23.2 -25.0 -21.8 -24.6	14M 70M 160M 410M	143000 50000 50000 50000
SICK SICK SICK SICK	SICK SICK SICK SICK	73.9 73.8 81.3 82.7	80.2 73.3 77.4 82.9	83.9 89.5 88.2 86.5	79.4 78.9 82.3 84.0		14M 70M 160M 410M	100000 10000 100000 100000
SICK SICK SICK SICK	SNLI SNLI SNLI SNLI	49.8 43.5 54.9 58.7	1.5 0.2 1.0 0.4	84.0 92.8 90.8 90.0	45.1 45.5 48.9 49.7	-34.3 -33.5 -33.4 -33.3	14M 70M 160M 410M	143000 100000 100000 50000

All models share the same basic architecture, but we test four different model sizes. Multiple pre-train checkpoints were tested but only the best-performing model of each size is shown here (see Figure 2 for those results). The Δ column marks the difference between the cross-domain and in-domain macro-average recall. For comparison with encoder models, see Talman and Chatzikyriakidis (2019, Table 4).

For the **zero-shot** models (pre-trained but with no NLI fine-tuning), we observe essentially chance performance (33.3% macro average recall) on SNLI for smaller models and slightly above chance performance for the largest model (38.4% macro average recall). The middle two zero-shot models have better than chance performance on SICK. While the performance is still very poor, the 160M parameter model performs 4.3 percentage points above chance with the full pre-training set. One possible explanation for this discrepancy is that the notion of entailment encoded in SICK is more closely aligned with what is evoked by the combination of Pythia's pre-training data and the NLI prompt we supplied. Curiously, the 70M parameter zero-shot model performs best after 50K pre-training sets and reverts to chance performance after seeing the whole pre-training set; the 410M parameter model's development set performance is highest after 10K training steps and degrades after that.

**In-domain** performance is generally quite good, especially for the largest models, though still a few percentage points below what is possible to achieve with off-the-shelf transformer encoder models. Models with more parameters generally perform better and pre-training data makes a difference, but performance flattens off or even degrades after 50K steps. This again points to the possibility that there is a discrepancy between the notion of entailment encoded in the pre-training data versus the NLI data, especially for SNLI.

**Cross-domain** performance drops significantly in both directions, which is compatible with the hypothesis that SNLI and SICK encode different notions of entailment, but see Section 6.4 for further discussion. Notably, the SICK performance of SNLI-trained models is significantly better than observed by Talman and Chatzikyriakidis (2019), which suggests that generative models may resist over-fitting more than encoder models, resulting in better cross-domain generalization. The performance of models trained on SICK and tested on SNLI is on a par with that reported by Kalouli et al. (2023) for BERT and RoBERTa. We find no evidence that the model's parameter count improves cross-domain generalization for the scale of models tested here.

### 6.4 Discussion

Talman and Chatzikyriakidis (2019) found that both transformer-encoder and recurrent models fine-tuned on SNLI (Bowman et al., 2015) and tested on SICK (Marelli et al., 2014) experience a drop in accuracy of about 30 percentage points (31.9 for ELMo and 33.5 for BERT-Base). In the same scenario, we found that the 160M Pythia model, which is closest in size to BERT-base, saw a somewhat more modest drop in accuracy of 21.8 percentage points. There are at least three components that could plausibly contribute to this poor cross-domain performance of the models: poor linguistic generalization, annotation artifacts, and poor inferential generalization. We address these in order below.

#### 6.4.1 Linguistic differences

The language of SICK and SNLI is broadly similar, both in terms of the source of the premises (as detailed in Section 6.2) as well as in terms of descriptive statistics (Tsuchiya, 2018). A basic quantitative analysis showing that roughly 20% of the SICK test pairs have at least one sentence in common with an SNLI training pair. We attempt to further separate linguistic from inferential cross-domain generalization, by separately reporting results for three different subsets of the SICK test set, for which some of the sentences occur also in the SNLI training set. Cross-domain performance on SICK pairs for which one of the sentences occur in some SNLI training set is largely comparable to the performance on the full SICK test set (Appendix Table 1).^17^ If failure of linguistic generalization were responsible for the SNLI–SICK cross-domain performance drop, then the performance drop would be less pronounced on the overlapping subset. This suggests that failure of linguistic generalization is less likely to alone account for the SNLI–SICK cross-domain performance drop.

The subset of SICK test pairs where the hypothesis also appear as an hypothesis in the SNLI train set exhibit significantly worse cross-domain performance (Appendix Table 2).^18^ One possible explanation is that for a fair share of these pairs the model has seen the hypothesis many times in training with a range of different labels attached to it. For about 36% of these pairs, the SICK gold label for the pair is not among the gold labels of SNLI training items with the same hypothesis. A concrete example is SICK item 1281, whose hypothesis appears in 47 SNLI train pairs. That SICK pair is labeled *C*, while one of the corresponding SNLI pairs is labeled *N* and the remaining 46 SNLI pairs are labeled *E*. For the given hypothesis, the premise varies much more than the label, which could cause the model to simply disregard the premise as in a hypothesis-only model.

#### 6.4.2 Annotation artifacts

An NLI training item typically contains a premise–hypothesis pair along with a label, and there are therefore three interrelations between these components than may contain both statistical information that are conducive to proper inferences, as well as biases that otherwise contribute to the predictions of a model. We distinguish between (1) premise–label, (2) hypothesis–label, and (3) premise–hypothesis relations. The first type of relations contain neither inferential information (since the hypothesis is missing) nor biases that are useful to models (Potts, 2019; Baas, 2021), and can therefore be ignored. The second type contain no proper inferential information, but may suffer from a “hypothesis-only” bias that is likely based on annotation artifacts such as “give-away” words in the hypotheses (Gururangan et al., 2018; Poliak et al., 2018; Tsuchiya, 2018). The third type of relations contain the proper inferential relationship, as well as biases that depend on some non-inferential relationship between premise and hypothesis, such as lexical overlap or other syntactic clues taking both the premise and hypothesis into account (McCoy et al., 2019).^19^

The cross-domain deltas for the full models can therefore in general be explained by a combination of (1) linguistic differences previously discussed, and differences in the (2) hypothesis–label relations and (3) premise–hypothesis relations. Note that the learned premise–hypothesis relations contain both the interesting inferential relationship as well as any lexical-overlap (or similar) biases, and these two components cannot be further experimentally separated in the current setting.

A hypothesis-only model effectively ignores the inference component of NLI, and only relies on annotation artifacts or other statistical patterns over hypothesis–label pairs in classifying the test set. The amount of annotation bias in a dataset can be estimated by the difference in performance of a hypothesis-only model and a majority class baseline (Poliak et al., 2018).^20^ Following a similar methodology, Belinkov et al. (2019) estimate the degree of similarity of biases in different datasets by measuring cross-domain performance for hypothesis-only models. It is well known that SNLI is rife with annotation artifacts, that make hypothesis-only models perform surprisingly well (Gururangan et al., 2018; Poliak et al., 2018). By contrast, SICK does not seem to suffer from such artifacts. This is likely due to the procedural way in which the hypotheses were generated, and it is shown by Poliak et al. (2018); Tsuchiya (2018) that the hypothesis-only baseline for this dataset is essentially the majority-class baseline. This suggests that the systematic failure of cross-domain performance in both directions cannot be explained entirely by hypothesis–label annotation artifacts in the data.

The results of our hypothesis-only cross-domain experiments confirms this (Appendix Table 4). Since the hypothesis-only models ignore the premise–hypothesis relation, and therefore the inferential relationship as well as any premise–hypothesis biases, the cross-domain deltas for such models can not be explained by poor inferential generalization nor mismatching lexical-overlap biases. Hence, the closer the two deltas are two each other, the more of the full model performance drop can instead be attributed to linguistic differences and differences in the premise–hypothesis relations. When we compare the corresponding cross-domain deltas we find that the performance drop is less pronounced for the cross-domain hypothesis-only models than it is for the cross-domain full models in Table 2. This means that having access to the premise–hypothesis relation impedes cross-domain generalization, a finding that it as least consistent with the hypothesis that SNLI and SICK encode different notions of entailment. An alternative explanation is that any premise–hypothesis biases differ between SNLI and SICK.

The difference between full-model cross-domain delta and hypothesis-only cross-domain delta estimates the degree to which cross-domain performance drop can be attributed to differences in premise–hypothesis relations. These differences for models trained on SNLI and tested on SICK are less drastic (in the range 1.3–8.5) than they are in the other direction (22.5–25.5). This suggests that less of the cross-domain performance drop from SNLI to SICK can be explained by poor inferential generalization together with differences in premise–hypothesis bias.

Taken together with the fact that the cross-domain performance drop for full models trained on SNLI is also less pronounced than for those trained on SICK, this suggests the premise–hypothesis relation encoded by SICK is more restrictive than the one encoded by SNLI: It would seem to be easier to learn SICK premise–hypothesis relations from SNLI, than vice versa. This too is compatible with the hypothesis that SNLI and SICK encode different notions of entailment, but could also be explained by any lexical-overlap (or similar) bias learned from of SNLI being more applicable to SICK, than vice versa.

#### 6.4.3 Inferential differences

The method by which inference relation labels were obtained differs significantly between the two datasets, suggesting that the respective dataset authors could be targeting different notions of entailment. As with SNLI, inference label descriptions in the SICK annotation instructions refer to *truth* and *falsity*, but they omit the explicit modal language found in the SNLI instructions (see Section 2.2):

- A is true, then B is true[…]
- If A is true, then B cannot be said to be true or false[…]
- If A is true, then B is false[…] (Marelli et al., 2014, p. 220)

Secondly, there is a significant procedural difference in how the items are constructed — in SNLI the annotator writes a hypothesis with a particular label in mind, whereas in SICK the annotator determines the label for a pair of sentences having been independently generated following a strict regimen.

Kalouli et al. (2019) point out that the SICK annotation process did not sufficiently clarify the role of coreference in relation to distinguishing between the *N* and *C* labels: For two sentences to contradict each other, there must be coreference between them (see also de Marneffe et al., 2008). By contrast, this fact is explicitly addressed by the SNLI instructions (Kalouli et al., 2019). Pairs that are not logical contradictions per se, may be conceived of as contradictions under the supposition that the referring expressions corefer, as is often assumed to be the case in SNLI with the premises and hypotheses taken to be (sufficiently exhaustive) image captions (see also Section 4.4). Hence sentence pairs that are labeled *N* when accurately following the SICK instructions may instead be labeled *C* according to the SNLI instructions, which implies that the SICK notion of contradiction is more restrictive than that of SNLI, while the SNLI neutral is more restrictive than that of SICK.

The label-separated accuracies in Table 2 and Appendix Table 4 show that models trained on SICK consistently perform very poorly in predicting the SNLI *C* label; this being the most pronounced cross-domain performance drop (−82.5 in the worst case). This aligns with the observation that SICK contradiction is more restrictive than SNLI contradiction: A model trained on the narrower notion of SICK's is unlikely to be able to generalize to the wider SNLI notion since no *C* pairs of the right kind are in the training data. Conversely, for models trained on SNLI the largest cross-domain performance drop is for the SICK *N* label; again consistent with the observation that the SNLI notion of neutral is the more restrictive.

These theoretical and experimental observations are consistent with the annotations of the only two sentence pairs that occur in both SNLI and SICK:

- “A baby is laughing”/“A baby is crying”;
- “A man is playing the drums”/“A woman is playing the drums”.

They are both in the respective training set, and they are both labeled *N* in SICK, but *C* in SNLI. These annotations are correct according to the respective annotation instructions. Hence the abstract consequence relations generated by the respective datasets are explictly different on these instances (even though the sample size is extremely small) along the lines of what Kalouli et al. (2023) call logical vs. common-sense reasoning. All in all, this points to that the poor cross-domain generalization can not in its entirety be explained by linguistic differences and annotation artifacts, but that differences in the inference relations encoded by the respective dataset do influence cross-domain generalization to some degree.

## 7 Conclusion

In Section 2.1, we suggested that the role of NLI as a diagnostic for generalized NLU depends on a close theoretical connection between linguistic meaning and inference. Our experiments in Section 6 contribute to a body of evidence that different NLI datasets encode different notions of inference. Which notion of inference, then, is the one with close ties to meaning? Another consistent result across NLI experiments points to the value of pre-training data across different NLI datasets. If we believe that (1) training a model for next-word (or masked-word) prediction results in internal representations that reflect *linguistic meaning* and (2) at least some of the performance gains of pre-trained models comes from improvements in inferential (and not only linguistic) generalization, then it would seem that *all* of these notions of inference have a close connection to meaning. Conversely, this would suggest that, even in ideal circumstances, a given NLI dataset can be expected to have limitations with respect to the aspects of linguistic meaning it can be expected to evaluate.

Other limitations of NLI as a method for evaluating NLU have to do with deviations from the evaluation pipeline suggested by the theoretical picture summarized in Section 2.1. In particular:

1. NLI/RTE datasets do not present arbitrary pairs of sentences from the target language, and exhibit certain regular biases that can be exploited by models. A clear demonstration of this limitation comes from the above-chance performance of *hypothesis-only baselines* on major NLI datasets — models provided with only the hypothesis of an NLI item perform well above chance on the SNLI and MultiNLI datasets (Gururangan et al., 2018). It is also notable that premise–hypothesis pairs are typically topically related, even when their entailment relation is *neutral*.
2. Standard practice involves fine-tuning the sentence encoder model when training a NLI/RTE classifier, which muddies the waters when attempting to determine the strength of the pre-trained representations — when training a classifier on top of an encoder model, it is important to at least compare with a scenario where the encoder is frozen.
3. Standard practice involves encoding a pair of sentences together, rather than classifying a pair of encoded sentences. This is important to allow the model to deal with contextual effects (such as anaphora between the premise and hypothesis), but it complicates matters somewhat if we are interested in the model's sentence-level NLU capability — or its ability to understand longer discourses, for that matter.

These deviations are fruitful areas of inquiry for understanding the shortcomings of NLI/RTE for assessing NLU in neural language models and how the situation may be different for certain kinds of neurosymbolic models.

Recommendations: We shouldn't rely on just one NLI dataset if we are interested in NLU. Different datasets may encode different notions of entailment, and all of these notions are likely to contribute to the inferentialist concept of meaning that underlies the connection between NLI and NLU. We must therefore distinguish between the external notion of consequence that we draw our logical intuitions from (“Contradictions are symmetric!”) and the internal notion of inference that is encoded in a dataset and ideally learned by the classifier. If we are using NLI as a probe for NLU, we should also pay close attention to the linguistic composition of the premises and hypotheses since this together with the external and internal consequence relations dictate for what fragment of the language and which types of meaning the model is evaluated.

## Data Availability

Publicly available datasets were analyzed in this study. This data can be found here: The datasets analyzed for this study can be found at the Stanford NLP Website (SNLI): https://nlp.stanford.edu/projects/snli/ and the Zenodo repository (SICK) https://zenodo.org/records/2787612. Pythia code and model weights are available on HuggingFace: https://huggingface.co/collections/EleutherAI/pythia-scaling-suite.

## Footnotes

1 See e.g., Venhuizen et al. (2022).

2 Section 3 discusses the difference between these two tasks in detail.

3 For a clear indication that these relations are not in the object language, it is enough to observe that the NLI labels do generally not occur in NLI premises or hypotheses.

4 See, e.g. Mendelson (2015), or any other modern textbook in mathematical logic, for the details of this view of formal languages and logical consequence.

5 Kalouli et al. (2023) makes a similar point regarding NLI classifiers bundling different notions of inference.

6 McCoy et al. (2020) distinguish between *in-distribution* vs. *out-of-distribution* generalizations, which is related to, but not exactly the same as our distinction between in-domain and cross-domain generalization. For example, the swapping of premise and hypothesis, as done by Wang et al. (2019b), would count as in-domain, out-of-distribution inferential generalization.

7 NLI datasets are available in a wide range of languages.

8 A similar point is raised by Bernardy and Chatzikyriakidis (2019).

9 A related point is made by McCoy et al. (2020) in their MNLI–HANS cross-domain evaluation of BERT models. They show that the order in which MNLI fine-tuning data is presented to BERT models may drastically affect out-of-distribution performance while keeping in-distribution performance more or less constant.

10 See Beltagy et al. (2016) and Kalouli et al. (2019) for a similar discussion.

11 Max pooling of final-layer token embeddings is a straight-forward technique, but unless specialized pre-training objectives are employed, there is no reason for it to result in a good sentence representation.

12 Prompting strategies that involve few-shot “in-context learning” can mitigate some of these problems by showing the model the correct answer format and exemplar inferences, but in-context learning has been shown to affect a model's internal weights in a way that is similar to fine-tuning (Oswald et al., 2023), so the trade-off remains.

13 In fact, the sole decoder-only model on the official SNLI leaderboard at https://nlp.stanford.edu/projects/snli/ is the Radford et al. (2018) GPT model. While no entries have been recorded to the leaderboard since 2021, which is likely partially explained by the declining interest in traditional NLI benchmarks, the few results on SNLI that have been published since then remain below those of the top encoder models.

14 Note that the 14M parameter model was added later and is not documented in Biderman et al. (2023). See https://huggingface.co/EleutherAI/pythia-14m for details.

15 These model sizes were chosen so that we can observe the effect of model size and compare with the encoder model results of Talman and Chatzikyriakidis (2019) and Kalouli et al. (2023). The 160M parameter model is comparable in size to BERT-base and RoBERTa-base, used in those experiments. The 410M parameter model is comparable in size to SOTA NLI models, such as that of Wang et al. (2021). Although better results are certain to be obtained with commercial LLMs, smaller open-source generative models are still scientifically and commercially relevant due to their cost efficiency, and the possibility for fine-tuning.

16 For fine-tuning, we used the SFT trainer implementation from the TRL Python library (von Werra et al., 2020). For parameter optimization we used the AdamW optimizer (Loshchilov and Hutter, 2019) with default hyperparameters (β_1_ = 0.9, β_2_ = 0.999, ϵ = 1e − 8) and an initial learning rate of 5e − 5.

17 We did not make a similar comparison between SICK training and SNLI test, since the proportion of such overlapping pairs were a lot smaller due to the relatively larger size of SNLI.

18 The subset of SICK test pairs that share their premise with an SNLI pair show better cross-domain performance, due to a drastic increase in recall on the *C* label. This subset is a lot smaller than the others, and results remain inconclusive.

19 McCoy et al. (2019) distinguish three types of biases (constituent, subsequence, lexical overlap), but also note that lexical overlap is the wider category that includes the two other. Therefore we focus our discussion on lexical overlap in the sequel.

20 Tsuchiya (2018) proposes as a more fine-grained measure the difference between model performance on the subset of pairs for which the hypothesis-only classification is correct and performance on the remaining sentence pairs. In mild contrast to this, a recent preprint by Phan (2026) suggests that an attempt to debias SNLI by ignoring sentence pairs with high hypothesis-only confidence also removes useful training signal.

## References

[B1] Agirre E. Cer D. Diab M. Gonzalez-Agirre A. (2012). “SemEval-2012 task 6: a pilot on semantic textual similarity,” in **SEM 2012: The First Joint Conference on Lexical and Computational Semantics*- *Volume 1: Proceedings of the main conference and the shared task, and Volume 2: Proceedings of the Sixth International Workshop on Semantic Evaluation (SemEval 2012)*, eds. E. Agirre, J. Bos, M. Diab, S. Manandhar, Y. Marton, and D. Yuret (Montréal: Association for Computational Linguistics), 385–393.

[B2] Baas A. (2021). Mitigating Bias in the SNLI Dataset (Bachelor's thesis). Utrecht University, Utrecht, The Netherlands. Available online at: https://studenttheses.uu.nl/handle/20.500.12932/40692

[B3] Belinkov Y. Poliak A. Shieber S. Van Durme B. Rush A. (2019). “Don't take the premise for granted: mitigating artifacts in Natural Language Inference,” in em Proceedings of the 57th Annual Meeting of the Association for Computational Linguistics, eds. A. Korhonen, D. Traum, and L. Màrquez (Florence: Association for Computational Linguistics), 877–891.

[B4] Beltagy I. Roller S. Cheng P. Erk K. Mooney R. J. (2016). Representing meaning with a combination of logical and distributional models. Comput. Linguist. 42, 763–808. doi: 10.1162/COLI_a_00266

[B5] Benayas A. Sicilia M. A. Mora-Cantallops M. (2025). A comparative analysis of encoder only and decoder only models in intent classification and sentiment analysis: navigating the trade-offs in model size and performance. Lang. Resour. Evaluat. 59, 2007–2030. doi: 10.1007/s10579-024-09796-y

[B6] Bernardy J. Chatzikyriakidis S. (2019). “What kind of natural language inference are NLP systems learning: Is this enough?,” in Proceedings of the 11th International Conference on Agents and Artificial Intelligence - *Volume 2: NLPinAI* (Set–bal: INSTICC, SciTePress), 919–931.

[B7] Biderman S. Schoelkopf H. Anthony Q. Bradley H. O'Brien K. Hallahan E. . (2023). Pythia: A Suite for Analyzing Large Language Models Across Training and Scaling. arXiv Preprint arXiv:2304.01373.

[B8] Blanck R. Noble B. Chatzikyriakidis S. (2025). “Reverse-engineering NLI: A study of the meta-inferential properties of natural language inference,” in Second Conference on Language Modeling. Montreal, QC: Palais des Congrès. 1–19.

[B9] Bos J. Markert K. (2005). “Recognising textual entailment with logical inference,” in Proceedings of Human Language Technology Conference and Conference on Empirical Methods in Natural Language Processing, eds. R. Mooney, C. Brew, L. F., Chien, and K. Kirchhoff (Vancouver, British Columbia: Association for Computational Linguistics), 628–635.

[B10] Bowman S. R. (2016). Modeling natural language semantics in learned representations (PhD thesis). Stanford University, Stanford, CA, United States.

[B11] Bowman S. R. Angeli G. Potts C. Manning C. D. (2015). “A large annotated corpus for learning natural language inference,” in Proceedings of the 2015 Conference on Empirical Methods in Natural Language Processing, eds. L. Màrquez, C. Callison-Burch, and J. Su (Lisbon: Association for Computational Linguistics), 632–642.

[B12] Brandom R. (2007). Inferentialism and some of its challenges. Philosophy Phenomenol. Res. 74, 651–676. doi: 10.1111/j.1933-1592.2007.00044.x

[B13] Brown T. B. Mann B. Ryder N. Subbiah M. Kaplan J. Dhariwal P. . (2020). Language Models are Few-Shot Learners.

[B14] Chen D. Dolan W. (2011). “Collecting highly parallel data for paraphrase evaluation,” in Proceedings of the 49th Annual Meeting of the Association for Computational Linguistics: Human Language Technologies, eds. D. Lin, Y. Matsumoto, and R. Mihalcea (Portland, Oregon: Association for Computational Linguistics), 190–200.

[B15] Chen X. Ye J. Zu C. Xu N. Zheng R. Peng M. . (2023). How Robust is GPT-3.5 to Predecessors? A Comprehensive Study on Language Understanding Tasks. arXiv Preprint arXiv:2303.00293.

[B16] Condoravdi C. Crouch D. de Paiva V. Stolle R. Bobrow D. G. (2003). “Entailment, intensionality and text understanding,” in Proceedings of the HLT-NAACL 2003 Workshop on Text Meaning (Stroudsburg, PA: Association for Computational Linguistics) 38–45.

[B17] Conneau A. Kiela D. Schwenk H. Barrault L. Bordes A. (2017). “Supervised learning of universal sentence representations from natural language inference data,” in Proceedings of the 2017 Conference on Empirical Methods in Natural Language Processing, eds. M. Palmer, R. Hwa, and S. Riedel (Copenhagen: Association for Computational Linguistics), 670–680.

[B18] Conneau A. Rinott R. Lample G. Williams A. Bowman S. Schwenk H. . (2018). “XNLI: Evaluating cross-lingual sentence representations,” in Proceedings of the 2018 Conference on Empirical Methods in Natural Language Processing, eds. E. Riloff, D. Chiang, J. Hockenmaier, and J. Tsujii (Brussels: Association for Computational Linguistics), 2475–2485.

[B19] Dagan I. Glickman O. Magnini B. (2006). “The PASCAL Recognising Textual Entailment challenge,” in Machine Learning Challenges. Evaluating Predictive Uncertainty, Visual Object Classification, and Recognising Tectual Entailment, J. Qui nonero-Candela, I. Dagan, B. Magnini, and F. d'Alché Buc (Berlin: Springer Berlin Heidelberg), 177–190.

[B20] Davidson D. (1967). Truth and meaning. Synthese 17, 304–323. doi: 10.1007/BF00485035

[B21] de Marneffe M.-C. Rafferty A. N. Manning C. D. (2008). “Finding contradictions in text,” in Proceedings of ACL-08: HLT, eds. J. D. Moore, S. Teufel, J. Allan, and S. Furui (Columbus, Ohio: Association for Computational Linguistics), 1039–1047.

[B22] Devlin J. Chang M.-W. Lee K. Toutanova K. (2019). “BERT: Pre-training of Deep Bidirectional Transformers for Language Understanding,” in Proceedings of the 2019 Conference of the North American Chapter of the Association for Computational Linguistics: Human Language Technologies, Volume 1 (Long and Short Papers) (Minneapolis, Minnesota: Association for Computational Linguistics), 4171–4186.

[B23] Font J. M. (2016). Abstract Algebraic Logic: An Introductory Textbook, volume 60 of Mathematical Logic and Foundations. London: College Publications.

[B24] Gao L. Biderman S. Black S. Golding L. Hoppe T. Foster C. . (2020). The Pile: An 800gb Dataset of Diverse Text for Language Modeling. arXiv Preprint arXiv:2101.00027.

[B25] Goldberg Y. (2017). Neural Network Methods for Natural Language Processing. Synthesis Lectures on Human Language Technologies. Cham: Springer.

[B26] Gubelmann R. Katis I. Niklaus C. Handschuh S. (2024). Capturing the varieties of natural language inference: a systematic survey of existing datasets and two novel benchmarks. J. Logic Lang. Inform. 33, 21–48. doi: 10.1007/s10849-023-09410-4

[B27] Gubelmann R. Niklaus C. Handschuh S. (2022). “A philosophically-informed contribution to the generalization problem of neural natural language inference: Shallow heuristics, bias, and the varieties of inference,” in Proceedings of the 3rd Natural Logic Meets Machine Learning Workshop (NALOMA III), eds. A.-L. Kalouli, and S. Chatzikyriakidis (Galway: Association for Computational Linguistics), 38–50.

[B28] Gururangan S. Swayamdipta S. Levy O. Schwartz R. Bowman S. Smith N. A. (2018). “Annotation artifacts in natural language inference data,” in Proceedings of the 2018 Conference of the North American Chapter of the Association for Computational Linguistics: Human Language Technologies, Volume 2 (Short Papers) (Galway: Association for Computational Linguistics), 107–112.

[B29] Hodosh M. Young P. Hockenmaier J. (2013). Framing image description as a ranking task: Data, models and evaluation metrics. J. Artif. Intellig. Res. 47, 853–899. doi: 10.1613/jair.3994

[B30] Incurvati L. Schlöder J. J. (2024). “Inferentialism,” in Reasoning with Attitude: Foundations and Applications of Inferential Expressivism (Oxford: Oxford University Press), 35–62.

[B31] Kalouli A.-L. Buis A. Real L. Palmer M. de Paiva V. (2019). “Explaining simple natural language inference,” in Proceedings of the 13th Linguistic Annotation Workshop, eds. A. Friedrich, D. Zeyrek, and J. Hoek (FlorenceL: Association for Computational Linguistics), 132–143.

[B32] Kalouli A.-L. Hu H. Webb A. F. Moss L. S. de Paiva V. (2023). Curing the SICK and other NLI maladies. Comput. Linguist. 49, 199–243. doi: 10.1162/coli_a_00465

[B33] Kassner N. Tafjord O. Schütze H. Clark P. (2021). “BeliefBank: Adding memory to a pre-trained language model for a systematic notion of belief,” in Proceedings of the 2021 Conference on Empirical Methods in Natural Language Processing, M.-F. Moens, X. Huang, L. Specia, and S. W.-t. Yih (Online and Punta Cana: Association for Computational Linguistics), 8849–8861.

[B34] Li H. Zhang Y. Koto F. Yang Y. Zhao H. Gong Y. . (2024). “CMMLU: Measuring massive multitask language understanding in Chinese,” in Findings of the Association for Computational Linguistics: ACL 2024, eds. L.-W. Ku, A. Martins, and V. Srikumar (Bangkok: Association for Computational Linguistics), 11260–11285.

[B35] Li T. Gupta V. Mehta M. Srikumar V. (2019). “A logic-driven framework for consistency of neural models,” in Proceedings of the 2019 Conference on Empirical Methods in Natural Language Processing and the 9th International Joint Conference on Natural Language Processing (EMNLP-IJCNLP), eds. K. Inui, J. Jiang, V. Ng, and X. Wan (Hong Kong: Association for Computational Linguistics), 3924–3935.

[B36] Liu H. Ning R. Teng Z. Liu J. Zhou Q. Zhang Y. (2023). Evaluating the logical reasoning ability of ChatGPT and GPT-4. arXiv Preprint arXiv:2304.03439.

[B37] Liu Y. Ott M. Goyal N. Du J. Joshi M. Chen D. . (2019). Roberta: A Robustly Optimized Bert Pretraining Approach.

[B38] Loshchilov I. Hutter F. (2019). Decoupled Weight Decay Regularization. arXiv Preprint arXiv:1711.05101.

[B39] MacCartney B. (2009). Natural language inference (Ph.d. thesis). Stanford University, Stanford, CA, United States.

[B40] MacCartney B. Manning C. D. (2014). “Natural logic and natural language inference,” in Computing Meaning: Volume 4, eds. H. Bunt, J. Bos, and S. Pulman (Springer: Dordrecht), 129–147.

[B41] Madaan L. Esiobu D. Stenetorp P. Plank B. Hupkes D. (2025). “Lost in inference: Rediscovering the role of natural language inference for large language models,” in Proceedings of the 2025 Conference of the Nations of the Americas Chapter of the Association for Computational Linguistics: Human Language Technologies (Volume 1: Long Papers), L. Chiruzzo, A. Ritter, and L. Wang (Albuquerque: Association for Computational Linguistics), 9229–9242.

[B42] Marelli M. Menini S. Baroni M. Bentivogli L. Bernardi R. Zamparelli R. (2014). “A SICK cure for the evaluation of compositional distributional semantic models,” in Proceedings of the Ninth International Conference on Language Resources and Evaluation (LREC'14), N. Calzolari, K. Choukri, T. Declerck, H. Loftsson, B. Maegaard, J. Mariani, A. Moreno, J. Odijk, and S. Piperidis (Reykjavik: European Language Resources Association (ELRA)), 216–223.

[B43] McCoy R. T. Min J. Linzen T. (2020). “BERTs of a feather do not generalize together: Large variability in generalization across models with similar test set performance,” in Proceedings of the Third BlackboxNLP Workshop on Analyzing and Interpreting Neural Networks for NLP, eds. A. Alishahi, Y. Belinkov, G. Chrupaa, D. Hupkes, Y. Pinter, and H. Sajjad (Stroudsburg, PA: Association for Computational Linguistics), 217–227.

[B44] McCoy R. T. Pavlick E. Linzen T. (2019). “Right for the wrong reasons: Diagnosing syntactic heuristics in natural language inference,” in Proceedings of the 57th Annual Meeting of the Association for Computational Linguistics, eds. A. Korhonen, D. Traum, and L. Màrquez (Florence: Association for Computational Linguistics).

[B45] Mendelson E. (2015). Introduction to Mathematical Logic. Boca Raton: CRC Press.

[B46] Minervini P. Riedel S. (2018). “Adversarially regularising neural NLI models to integrate logical background knowledge,” in Proceedings of the 22nd Conference on Computational Natural Language Learning, eds. A. Korhonen, and I. Titov (Brussels: Association for Computational Linguistics), 65–74.

[B47] Mitchell E. Noh J. Li S. Armstrong W. Agarwal A. Liu P. . (2022). “Enhancing self-consistency and performance of pre-trained language models through natural language inference,” in Proceedings of the 2022 Conference on Empirical Methods in Natural Language Processing, eds. Y. Goldberg, Z. Kozareva, and Y. Zhang (Abu Dhabi: Association for Computational Linguistics), 1754–1768.

[B48] Naik A. Ravichander A. Sadeh N. Rose C. Neubig G. (2018). “Stress test evaluation for natural language inference,” in Proceedings of the 27th International Conference on Computational Linguistics, eds. E. M. Bender, L. Derczynski, and P. Isabelle (Santa Fe, New Mexico: Association for Computational Linguistics), 2340–2353.

[B49] Nakamura M. Mashetty S. Parmar M. Varshney N. Baral C. (2023). “LogicAttack: Adversarial attacks for evaluating logical consistency of natural language inference,” in Findings of the Association for Computational Linguistics: EMNLP 2023, eds. H. Bouamor, J. Pino, and K. Bali (Singapore: Association for Computational Linguistics), 13322–13334.

[B50] Nangia N. Williams A. Lazaridou A. Bowman S. (2017). “The RepEval 2017 shared task: Multi-genre natural language inference with sentence representations,” in Proceedings of the 2nd Workshop on Evaluating Vector Space Representations for NLP, eds. S. Bowman, Y. Goldberg, F. Hill, A. Lazaridou, O. Levy, R. Reichart, and A. Søgaard (Copenhage: Association for Computational Linguistics), 1–10.

[B51] Nie Y. Williams A. Dinan E. Bansal M. Weston J. Kiela D. (2020). “Adversarial NLI: a new benchmark for natural language understanding,” in Proceedings of the 58th Annual Meeting of the Association for Computational Linguistics, eds. D. Jurafsky, J. Chai, N. Schluter, and J. Tetreault (Online: Association for Computational Linguistics), 4885–4901.

[B52] Oswald J. V. Niklasson E. Randazzo E. Sacramento J. Mordvintsev A. Zhmoginov A. . (2023). “Transformers learn in-context by gradient descent,” in Proceedings of the 40th International Conference on Machine Learning (New York: PMLR), 35151–35174.

[B53] Parcalabescu L. Frank A. (2024). “On measuring faithfulness or self-consistency of natural language explanations,” in Proceedings of the 62nd Annual Meeting of the Association for Computational Linguistics (Volume 1: Long Papers), eds. L.-W. Ku, A. Martins, and V. Srikumar (Bangkok: Association for Computational Linguistics), 6048–6089.

[B54] Peters M. E. Ruder S. Smith N. A. (2019). “To tune or not to tune? adapting pretrained representations to diverse tasks,” in Proceedings of the 4th Workshop on Representation Learning for NLP (RepL4NLP-2019) (Florence: Association for Computational Linguistics), 7–14.

[B55] Phan H. (2026). The challenge of debiasing NLI models: Why hypothesis-only confidence is insufficient. Available online at: https://engrxiv.org/preprint/view/6210/10223 (Accessed April 16, 2026).

[B56] Poliak A. Naradowsky J. Haldar A. Rudinger R. Van Durme B. (2018). “Hypothesis only baselines in natural language inference,” in Proceedings of the Seventh Joint Conference on Lexical and Computational Semantics, eds. M. Nissim, J. Berant, and A. Lenci (New Orleans, Louisiana: Association for Computational Linguistics), 180–191.

[B57] Potts C. (2019). Natural Language Inference. CS 224U Course Notes, Stanford University. Available online at: https://web.stanford.edu/class/cs224u/2019/materials/cs224u-2019-nli.pdf (Accessed May 11, 2026).

[B58] Qorib M. R. Moon G. Ng H. T. (2024). “Are decoder-only language models better than encoder-only language models in understanding word meaning?,” in Findings of the Association for Computational Linguistics: ACL 2024, eds. L.-W. Ku, A. Martins, and V. Srikumar (Bangkok: Association for Computational Linguistics), 16339–16347.

[B59] Radford A. Narasimhan K. Salimans T. Sutskever I. (2018). Improving Language Understanding by Generative Pre-Training (San Francisco, CA: OpenAI).

[B60] Srikanth N. Rudinger R. (2025). “NLI under the microscope: what atomic hypothesis decomposition reveals,” in Proceedings of the 2025 Conference of the Nations of the Americas Chapter of the Association for Computational Linguistics: Human Language Technologies (Volume 1: Long Papers), eds. L. Chiruzzo, A. Ritter, and L. Wang (Albuquerque, NM: Association for Computational Linguistics), 2574–2589.

[B61] Steinberger F. Murzi J. (2017). “Inferentialism,” in Blackwell Companion to Philosophy of Language, eds. S. Florian, and M. Julien (Malden: Blackwell), 197–224.

[B62] Sun Z. Fan C. Han Q. Sun X. Meng Y. Wu F. . (2020). Self-Explaining Structures Improve NLP Models. arXiv Preprint arXiv:2012.01786.

[B63] TAC2009 (2009). RTE-5 Main Task Guidelines. Available online at: https://tac.nist.gov/2009/RTE/RTE5_Main_Guidelines.pdf (Accessed October 21, 2025).

[B64] Talman A. Chatzikyriakidis S. (2019). “Testing the generalization power of neural network models across NLI benchmarks,” in Proceedings of the 2019 ACL Workshop BlackboxNLP: Analyzing and Interpreting Neural Networks for NLP, eds. T. Linzen, G. Chrupała, Y. Belinkov, and D. Hupkes (Florence: Association for Computational Linguistics), 85–94.

[B65] Tarski A. (1986 [1933]). “The concept of truth in formalized languages,” in Logic, Semantics, Metamathematics, ed. J. Corcoran (Hackett, Indianapolis: expanded version, English translation by J. H. Woodger), 152–278.

[B66] Tsuchiya M. (2018). “Performance impact caused by hidden bias of training data for Recognizing Textual Entailment,” in Proceedings of the Eleventh International Conference on Language Resources and Evaluation (LREC 2018), eds. N. Calzolari, K. Choukri, C. Cieri, T. Declerck, S. Goggi, K. Hasida, et al. (Miyazaki: European Language Resources Association (ELRA)).

[B67] Venhuizen N. J. Hendriks P. Crocker M. W. Brouwer H. (2022). Distributional formal semantics. Inform. Comput. 287:104763. doi: 10.1016/j.ic.2021.104763

[B68] von Werra L. Belkada Y. Tunstall L. Beeching E. Thrush T. Lambert N. . (2020). TRL: Transformers Reinforcement Learning. Available online at: https://github.com/huggingface/trl (Accessed January 26, 2026).

[B69] Wang A. Pruksachatkun Y. Nangia N. Singh A. Michael J. Hill F. . (2019a). “Superglue: a stickier benchmark for general-purpose language understanding systems,” in Advances in Neural Information Processing Systems, eds. H. Wallach, H. Larochelle, A. Beygelzimer, F. d' Alché-Buc, E. Fox, and R. Garnett (Red Hook: Curran Associates, Inc.).

[B70] Wang A. Singh A. Michael J. Hill F. Levy O. Bowman S. (2018). “GLUE: A multi-task benchmark and analysis platform for natural language understanding,” in Proceedings of the 2018 EMNLP Workshop BlackboxNLP: Analyzing and Interpreting Neural Networks for NLP, eds. T. Linzen, G. Chrupała, and A. Alishahi (Brussels: Association for Computational Linguistics), 353–355.

[B71] Wang H. Sun D. Xing E. P. (2019b). “What if we simply swap the two text fragments? A straightforward yet effective way to test the robustness of methods to confounding signals in nature language inference tasks,” in Proceedings of the Thirty-Third AAAI Conference on Artificial Intelligence and Thirty-First Innovative Applications of Artificial Intelligence Conference and Ninth AAAI Symposium on Educational Advances in Artificial Intelligence, AAAI'19/IAAI'19/EAAI'19. Singapore: AAAI Press.

[B72] Wang S. Fang H. Khabsa M. Mao H. Ma H. (2021). Entailment as Few-Shot Learner.

[B73] Weller O. Ricci K. Marone M. Chaffin A. Lawrie D. Durme B. V. (2026). “Seq vs Seq: An open suite of paired encoders and decoders,” in The Fourteenth International Conference on Learning Representations. Available online at: https://openreview.net/forum?id=z5Mn8Rxi3l (Accessed May 6, 2026).

[B74] Williams A. Nangia N. Bowman S. (2018). “A broad-coverage challenge corpus for sentence understanding through inference,” in Proceedings of the 2018 Conference of the North American Chapter of the Association for Computational Linguistics: Human Language Technologies, Volume 1 (Long Papers), eds. M. Walker, H. Ji, and A. Stent (New Orleans, Louisiana: Association for Computational Linguistics), 1112–1122.

[B75] Wu W. Last M. (2025). “Transitive self-consistency evaluation of NLI models without gold labels,” in Proceedings of the 2025 Conference on Empirical Methods in Natural Language Processing, eds. C. Christodoulopoulos, T. Chakraborty, C. Rose, and V. Peng (Suzhou: Association for Computational Linguistics), 22637–22653.

[B76] Ye J. Chen X. Xu N. Zu C. Shao Z. Liu S. . (2023). A Comprehensive Capability Analysis of GPT-3 and GPT-3.5 Series Models.

[B77] Young P. Lai A. Hodosh M. Hockenmaier J. (2014). From image descriptions to visual denotations: New similarity metrics for semantic inference over event descriptions. Trans. Assoc. Computat. Linguist. 2, 67–78. doi: 10.1162/tacl_a_00166

[B78] Zhang J. Huang Y. Liu S. Gao Y. Hu X. (2025). “Do BERT-like bidirectional models still perform better on text classification in the era of LLMs?,” in Findings of the Association for Computational Linguistics: EMNLP 2025, eds. C. Christodoulopoulos, T. Chakraborty, C. Rose, and V. Peng (Suzhou: Association for Computational Linguistics), 18980–18989.

