# Supplementary material for: Logic, inference, understanding: cross-domain generalization for generative language models
Source: Front Artif Intell. 2026 Jun 16;9:1800372. doi: 10.3389/frai.2026.1800372 (PMC13314620; doi:10.3389/frai.2026.1800372)
Supplement: Supplementary file 1 [file Supplementary_file_1.pdf]

1 OVERLAPPING DATA RESULTS

Results for SICK items that appear in the SNLI train set. The distribution of labels E/C/N for the full SICK test set is 28.6%/14.5%/56.8%.

Table 1. Subset of SICK results presented in Table 2 where either of the test sentences appear in the SNLI train set (N=997). Labels E/C/N are distributed 31.3%/12.6%/56.1% in this subset. The Δ column shows the differential between performance on the full test set (Table 2) and the restricted test set, with a positive differential indicating better performance on the restricted set.

Train	Test	E	C	N	macro avg.	Δ	Size	Steps
zero-shot	SICK	98.4	2.4	0.0	33.6	-0.3	14M	0
zero-shot	SICK	0.3	12.7	96.1	36.4	-1.4	70M	50000
zero-shot	SICK	75.6	11.1	21.6	36.1	-1.5	160M	143000
zero-shot	SICK	98.4	0.0	2.1	33.5	-0.9	410M	10000
SNLI	SICK	88.1	43.7	23.4	51.7	-0.9	14M	143000
SNLI	SICK	90.1	43.7	19.7	51.1	-3.2	70M	50000
SNLI	SICK	90.4	63.5	27.0	60.3	-0.0	160M	50000
SNLI	SICK	90.4	44.4	26.8	53.9	-2.2	410M	50000
SICK	SICK	73.1	82.5	88.4	81.3	+1.9	14M	100000
SICK	SICK	75.6	75.4	93.7	81.6	+2.7	70M	10000
SICK	SICK	83.7	84.9	93.4	87.3	+5.0	160M	100000
SICK	SICK	84.6	85.7	90.5	86.9	+2.9	410M	100000

Table 2. Subset of SICK results presented in Table 2 where the test hypothesis appears as a hypothesis in the SNLI train set (N=472). Labels E/C/N are distributed 37.5%/11.4%/51.1% in this subset. The Δ column shows the differential between performance on the full test set (Table 2) and the restricted test set, with a positive differential indicating better performance on the restricted set.

Train	Test	E	C	N	macro avg.	Δ	Size	Steps
zero-shot	SICK	98.9	0.0	0.0	33.0	-0.9	14M	0
zero-shot	SICK	0.0	9.3	95.9	35.0	-2.8	70M	50000
zero-shot	SICK	77.4	1.9	20.7	33.3	-4.3	160M	143000
zero-shot	SICK	98.3	0.0	2.1	33.5	-0.9	410M	10000
SNLI	SICK	92.1	13.0	14.5	39.9	-12.7	14M	143000
SNLI	SICK	93.2	11.1	11.6	38.6	-15.7	70M	50000
SNLI	SICK	96.0	48.1	16.2	53.5	-6.8	160M	50000
SNLI	SICK	94.9	9.3	22.0	42.1	-14.0	410M	50000
SICK	SICK	74.6	85.2	84.2	81.3	+1.9	14M	100000
SICK	SICK	80.2	75.9	93.8	83.3	+4.4	70M	10000
SICK	SICK	88.7	88.9	92.5	90.0	+7.7	160M	100000
SICK	SICK	89.8	87.0	90.5	89.1	+5.1	410M	100000

Table 3. Subset of SICK results presented in Table 2 where the test premise appears as a premise in the SNLI train set (N=196). Labels E/C/N are distributed 30.1%/11.7%/57.6% in this subset. The Δ column shows the differential between performance on the full test set (Table **??**) and the restricted test set, with a positive differential indicating better performance on the restricted set.

Train	Test	E	C	N	macro avg.	Δ	Size	Steps
zero-shot	SICK	100.0	0.0	0.0	33.3	-0.6	14M	0
zero-shot	SICK	0.0	17.4	98.2	38.5	+0.7	70M	50000
zero-shot	SICK	78.3	21.7	23.0	41.0	+3.4	160M	143000
zero-shot	SICK	98.3	0.0	2.7	33.7	-0.7	410M	10000
SNLI	SICK	81.7	91.3	24.8	65.9	+13.3	14M	143000
SNLI	SICK	88.3	95.7	20.4	68.1	+13.8	70M	50000
SNLI	SICK	86.7	95.7	28.3	70.2	+9.9	160M	50000
SNLI	SICK	83.3	95.7	29.2	69.4	+13.3	410M	50000
SICK	SICK	73.3	78.3	91.2	80.9	+1.5	14M	100000
SICK	SICK	70.0	78.3	92.0	80.1	+1.2	70M	10000
SICK	SICK	76.7	87.0	95.6	86.4	+4.1	160M	100000
SICK	SICK	81.7	82.6	89.4	84.6	+0.6	410M	100000

2 HYPOTHESIS-ONLY RESULTS

Table 4. Hypothesis-only results for the top-performing (in-domain) Pythia models of the 160M and 410M sizes. The Δ column shows the difference in macro average accuracy between the hypothesis-only and full models (Table 2). We also show the micro-average performance here for comparison with Poliak et al. (2018), but note that micro average is not comparable across test sets since the SNLI test set is essentially balanced while SICK is not. The majority class for SICK is *neutral* (56.9) while the majority class for SNLI is *entailment* (34.3).

Train	Test	E	C	N	macro avg.	Δ	micro avg.	Size	Steps
SNLI	SNLI	76.8	58.7	57.3	64.3	-17.8	64.5	160M	50000
SNLI	SICK	57.5	54.6	19.3	43.8	-16.5	35.3	160M	50000
SICK	SICK	17.9	30.9	81.6	43.5	-38.8	56.0	160M	100000
SICK	SNLI	8.5	3.4	94.8	35.6	-13.3	35.1	160M	100000
SNLI	SNLI	65.2	49.5	74.4	63.0	-17.7	63.0	410M	50000
SNLI	SICK	37.0	61.8	41.9	46.9	-9.2	43.4	410M	50000
SICK	SICK	86.9	27.9	19.9	44.9	-39.1	40.2	410M	50000
SICK	SNLI	87.4	2.6	9.4	33.1	-16.6	33.9	410M	50000
